# Cardiac rehabilitation and telemedicine (and COVID-19)

**DOI:** 10.1007/s12471-020-01473-3

**Published:** 2020-07-15

**Authors:** R. J. G. Peters

**Affiliations:** 1 Department of Cardiology, Amsterdam UMC, University of Amsterdam, Heart Center, Amsterdam Cardiovascular Sciences, Amsterdam, The Netherlands; 2 Department of Cardiology, Amsterdam UMC, Free University, Heart Center, Amsterdam Cardiovascular Sciences, Amsterdam, The Netherlands

Cardiac rehabilitation (CR) programmes reduce morbidity and mortality in cardiac patients, with effect sizes that are comparable to those of antiplatelet, lipid-lowering or blood pressure–lowering therapy. In addition, participation in CR programmes increases quality of life [1]. Current guidelines indicate a class 1A recommendation for CR [2, 3].

However, only a minority of cardiac patients participate in these programmes. This is related to limited capacity, to low referral rates (particularly in patients with chronic cardiac conditions) and to patient factors that include age, socioeconomic status and practical issues such as geographical distance [4]. Dropout from CR programmes is mainly related to these same factors. Dropout is associated with unfavourable outcomes, as reported by Sunamura et al. in this issue [5]. Premature termination of the CR programme represents a loss of benefits, a waste of resources and a waste of the limited capacity for CR. Patients who do complete a centre-based CR programme are commonly not offered follow-up coaching and support. Most programmes are limited to a duration of 12 weeks, after which a loss of effect on risk profiles and exercise capacity is to be expected.

Cardiac telerehabilitation (CTR) has the potential to overcome several of the barriers and limitations of the current centre-based CR. Patient volumes may be significantly greater, travel issues (including costs and pollution) are avoided, coaching and support can be personalised, CR can be provided in shorter sessions if appropriate, and support may be offered for longer periods of time. Individual health data, such as heart rate, can be assessed during normal daily activities, which allows personalised feedback and education by a healthcare professional.

The review article by Brouwers et al. in this issue outlines the numerous advantages of CTR [6]. As the authors point out, an initial centre-based introduction and evaluation appears appropriate, from both a safety and a psychological standpoint. Patients may be more motivated if they know the members of the professional team and may be more confident after initial testing and exercising at the rehabilitation centre. In addition, the (initial) company of other patients may be stimulating and motivating.

For a balanced view on CTR, potential disadvantages need to be considered. With home-based CR, the patient does not benefit from the environment of a professional CR centre, including the company of other patients who may provide peer support, the physical presence of healthcare professionals and the availability of equipment in case of complications. Fortunately, the risk of cardiac complications during activities of rehabilitation is very low. In a large observational study in France, the cardiac arrest rate was 1.3 per million patient-hours of exercise; neither fatal complications nor emergency defibrillations were reported [7].

CTR aligns perfectly with the recent societal measures that have been instituted to control the outbreak of the coronavirus disease (COVID-19). For patients with heart disease, CTR offers a significant additional benefit by limiting exposure to others during group meetings.
